# Patient characteristics associated with different types of prison TB: an epidemiological analysis of 921 TB cases diagnosed at an Ethiopian prison

**DOI:** 10.1186/s12890-021-01699-w

**Published:** 2021-10-27

**Authors:** Asmah Amirkhani, Maheen Humayun, Wen Ye, Yoseph Worku, Zhenhua Yang

**Affiliations:** 1 University of Michigan, M5124 SPH II, 1415 Washington Heights, Ann Arbor, MI 48109-2029 USA; 2 St. Paul’s Hospital Millennium Medical College, Swaziland St, Gulelle Sub-City, P.O. Box 40742, Addis Ababa, Ethiopia

**Keywords:** Tuberculosis, HIV, Prison TB, Epidemiology, Ethiopia

## Abstract

**Background:**

Despite incarcerated population being at an increased risk of tuberculosis (TB) and serving as a potential source of TB transmission for the general population, prison TB remains understudied. Given its adverse impact on progress towards TB elimination, World Health Organization (WHO) has identified prison TB research as a top priority to guide TB treatment/control interventions.

**Methods:**

We retrospectively analyzed 921 notified TB cases that were diagnosed at Kality Federal Prison, Ethiopia during 2009–2017. To assess trends of microbiologically confirmed pulmonary TB (PTB), extra-pulmonary TB (EPTB), and TB-HIV co-infection, an ecological analysis of aggregated cases was used to report trends over time. Additionally, we used multivariable log binomial regression to identify patient characteristics associated with microbiologically confirmed PTB, EPTB, and TB-HIV co-infection.

**Results:**

Microbiologically confirmed PTB proportion increased over time. Young age was identified as an important risk factor for EPTB (adjusted prevalence ratio [aPR] = 1.74, 95% CI 0.97, 3.13) while HIV coinfection was negatively associated with EPTB (aPR = 0.73, 95% CI 0.55, 0.97). While previous TB history was associated with a lower likelihood of EPTB (aPR = 0.42, 95% CI 0.25, 0.70), it was associated with an increased risk of TB-HIV coinfection (aPR = 1.37, 95% CI 1.10, 1.71). Clinically diagnosed PTB patients were more likely to have TB-HIV coinfection compared to microbiologically confirmed PTB patients (aPR = 1.32, 95% CI 1.02, 1.72).

**Conclusions:**

Increasing proportion of microbiologically confirmed PTB may suggest delayed access to treatment, severe disease and increased risk of intramural transmission. Associations with clinical/demographic factors varied for different types of TB and were not always consistent with what has been previously reported for the general population, necessitating the need to refocus prison TB control/treatment strategies based on context specific epidemiological factors.

**Supplementary Information:**

The online version contains supplementary material available at 10.1186/s12890-021-01699-w.

## Introduction

Despite tremendous global efforts towards tuberculosis (TB) elimination, TB remains one of the top ten causes of death and the most deadly infectious disease globally [1]. In 2019, TB occurred in 10 million people, killing 1.2 million HIV negative and 208,000 HIV positive TB patients [1]. It is a leading cause of death among HIV infected individuals, exacerbating the TB epidemic in HIV high burden countries [2].

Given these staggering numbers, global efforts led by the WHO have been intensified to tackle the TB epidemic but progress towards global TB elimination falls behind set targets in many high burden countries [3]. As countries move towards achieving the WHO’s End TB Strategy goals, it is important to realize that a one for all approach will not produce desired results, owing to country specific contextual and epidemiological factors [4].

Even aggregated national estimates may not reflect equitable improvements for all subpopulations within the country, especially those that are most vulnerable. Incarcerated population, which was targeted by the current study, experiences a much higher incidence of TB compared to the general population and is often overlooked when tabulating aggregated estimates [5, 6]. According to estimates from a systematic review, TB prevalence is 3.75–1000 times higher in prisons compared to the corresponding general population [7]. The increased TB occurrence in prisons has been attributed to a disproportionate representation of already high-risk persons such as the socio-economically disadvantaged, homeless and refugees in prisons [8, 9]. Further, the prison environment that is generally poorly ventilated and overcrowded provides a suitable setting for increased transmission [9]. Not only is the incarcerated population itself at an increased risk of TB infection and mortality, they also serve as a potential source of TB transmission for the civilian population through multiple portals of exit including staff, visitors, prison transfers and contact upon release [6, 10, 11]. Hence, TB outbreaks within prisons remain an imminent threat to the general population and progress towards global TB elimination.

Here, we report an epidemiological study of TB at the Kality Federal Prison, Ethiopia during 2009–2017. Ethiopia is a high burden country with respect to TB incidence, multidrug resistant (MDR) TB incidence and TB incidence among HIV infected individuals [1]. There is insufficient data available to track national prison TB estimates and inform prevention/control strategies within such high-risk settings in Subsaharan Africa where research has been limited to only a handful of prisons [5, 7]. WHO has identified prison TB epidemiological research aimed at evaluating prison TB prevalence and existing interventions as a top research priority to enhance equitable access to care and treatment [12]. Hence, by investigating prison specific TB epidemiology and over time epidemiological transitions, the present study aims to extend the knowledge base of prison TB and TB-HIV coinfection to guide the development of an effective TB control strategy in high burden settings.

## Methods

### Study design

We conducted an ecological study of tuberculosis for the period of 2009–2017 to assess trends related to microbiologically confirmed pulmonary TB (PTB), extra-pulmonary TB (EPTB), and TB-HIV co-infection. In addition, a cross-sectional analysis of patient characteristics associated with EPTB, microbiologically confirmed PTB (based on sputum smear microscopy or Gene Xpert assay) and TB-HIV co-infection was conducted using this closed cohort of TB patients.

### Study population

The study population included 921 adult (18 years or older) inmates of Kality Federal Prison diagnosed with TB during 2009–2017. Kality is a major prison in Ethiopia, holding approximately 4500 prisoners [13]. The inmates were enrolled for TB treatment at two health facilities under the Federal Prison Administration: a general hospital and a health center. These notified cases included new, relapse and previously treated TB patients.

### Data collection

Data was extracted from three TB Unit Registers that consolidated and recorded prison TB cases from the patients’ medical records. For cases that had missing data in the TB unit registers, medical records were referenced to collect additional data. Demographic and clinical variables including age, gender, year of diagnosis, TB type, previous history of TB, HIV sero-positivity and microbiological confirmation of PTB were collected for the purpose of this study. This study was approved by the University of Michigan Institutional Review Board—Health Science and Behavior Sciences, the St. Paul’s Hospital Millennium Medical College Institutional Review Board, and the Kality Prison Administration. All the analyses of the present study used de-identified data.

### Data analysis

Our time trend ecological analysis provided aggregated estimates for annually notified TB cases for the study period, 2009–2017. Further, annual TB cases were stratified by TB type (EPTB vs. PTB), PTB annual cases were stratified by microbiological confirmation of PTB (clinically diagnosed PTB vs. microbiologically confirmed PTB), and annual TB cases with a recorded HIV result were stratified by HIV sero-positivity (HIV sero-positive vs. HIV sero-negative). The percent proportion of EPTB and PTB among annual TB cases, the percent proportion of clinically diagnosed and microbiologically confirmed cases among annual PTB cases, and the percent proportion of HIV sero-positive cases among annual TB cases with a recorded HIV result were calculated.

TB disease manifests in different forms, each of which has varying implications for TB transmission, diagnosis and treatment. Hence, we separately assessed the association between these three outcomes (EPTB, microbiological confirmation of PTB, and TB-HIV co-infection) and demographic/clinical characteristics. Using log binomial regression, we report adjusted prevalence ratios (aPRs) with 95% confidence intervals instead of a logistic regression reporting odds ratios (ORs) as the outcome was not rare.

The first model assessed the association between TB type (EPTB vs. PTB) and patient characteristics including age group, previous TB history (yes vs. no), HIV sero-positivity (sero-positive vs. sero-negative) and year of diagnosis. The continuous age data was collapsed into 4 categories: 18–24, 25–44, 45–64, and 65+ years. Patients who were lost to follow up, failed treatment or relapsed after previous TB treatment were encoded as having a previous history of TB. TB type was dichotomized into PTB and EPTB categories by collapsing both clinically diagnosed PTB and microbiologically confirmed PTB as PTB.

The second model assessed the association between microbiological confirmation of PTB (microbiologically confirmed PTB vs clinically diagnosed PTB) and patient characteristics including age group, previous history of TB, HIV sero-positivity and year of diagnosis. All cases, but one, that received a microbiologically confirmed PTB diagnosis had a documented positive result for either Gene Xpert or sputum smear microscopy.

The last model assessed the association between HIV sero-positivity and patient characteristics including age group, previous history of TB, TB diagnosis (EPTB/clinically diagnosed PTB/microbiologically confirmed PTB) and year of diagnosis. All three models converged using log binomial regression. These three models excluded those above the age of 65 years due to small cell counts. All statistical analyses were performed using SAS version 9.4 (SAS Institute, Cary, NC, USA) and data visualization was performed using Excel in Microsoft Office.

## Results

### Demographic and clinical characteristics of study patients

The study prison admits both males and females, but the incarcerated population during our study period was predominantly male with only 2.1% of the study population representing women (Table 1). Younger individuals between 18 and 44 years represented 88.9% of the total cases. A majority (83.5%) of the cases in our study population had no record of prior TB history. The highest number of TB cases were recorded in 2016. Of the 921 reported cases of TB during the study period, 67.4% were PTB cases. Among PTB cases (n = 621), 60.9% of the cases were clinically diagnosed, 31.7% had a recorded positive sputum smear result and 7.2% had a recorded positive Gene Xpert result. A HIV test result was available for 84.4% (n = 778) of our final sample and 30.6% of these cases were HIV positive.

**Table 1:** Demographic and clinical characteristics of 921 tuberculosis (TB) cases at Kality Federal Prison, Ethiopia during 2009–2017

Characteristics	No. (%^a^)
Age	
18–24	345 (37.5)
25–44	473 (51.4)
45–64	82 (8.9)
65+	13 (1.4)
Unknown	8 (0.9)
Gender	
Male	902 (97.9)
Female	19 (2.1)
Previous history of TB	
No	769 (83.5)
Yes	123 (13.4)
Unknown	29 (3.1)
TB type	
PTB	621 (67.4)
EPTB	300 (32.6)
PTB diagnosis (n = 621)	
MC-Sputum smear positive	197 (31.7)
MC-Gene Xpert positive	45 (7.2)
MC-unknown	1 (0.2)
Clinically diagnosed	378 (60.9)
Year	
2009	73 (7.9)
2010	137 (14.9)
2011	118 (12.8)
2012	98 (10.6)
2013	90 (9.8)
2014	96 (10.4)
2015	96 (10.4)
2016	146 (15.9)
2017	67 (7.3)
HIV serological status	
Positive	238 (25.8)
Negative	540 (58.6)
Unknown	143 (15.5)

*MC* microbiologically confirmed

^a^Due to rounding, percentages may not add up to 100

### Trends of different types of TB

**Fig. 1:**
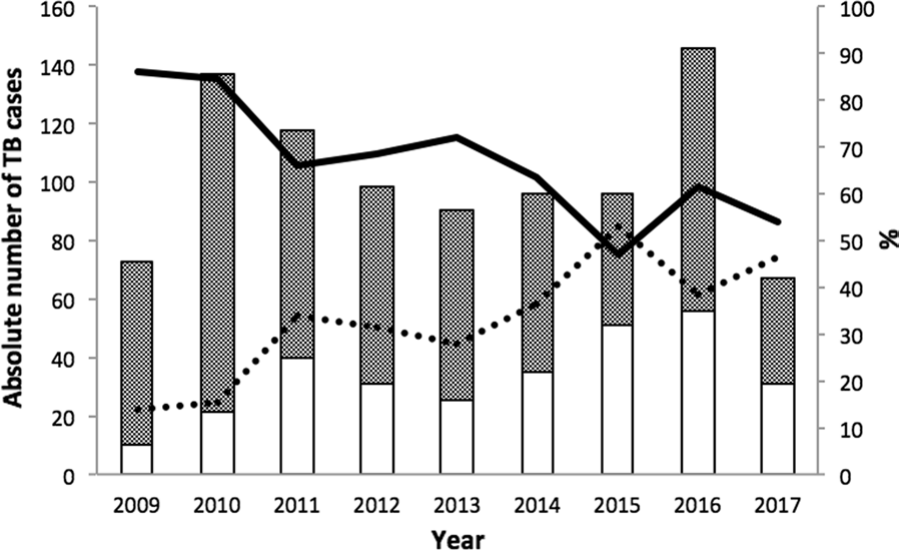
Extra-pulmonary TB/pulmonary TB trends at Kality Federal Prison, Ethiopia, during 2009–2017. The annual numbers of notified tuberculosis cases among Kality Federal prisoners (n = 921) during 2009–2017 are shown in the bar chart, stratified by type of tuberculosis (extra-pulmonary TB/pulmonary TB). The shaded bar represents pulmonary TB cases; the white bar represents extra-pulmonary TB. The line graph provides the proportion of pulmonary TB (solid line) and extra-pulmonary TB cases (dotted line), respectively

The annual number of notified TB cases diagnosed at Kality Federal Prison ranged between 67 and 146 cases during 2009–2017 (Fig. 1). During the entire study period, with the exception of 2015, the proportion of PTB among TB cases remained higher than that of EPTB. However, over time the proportion of EPTB increased from 13.7% in 2009 to 46.3% in 2017 while the proportion of PTB decreased from 86.3% in 2009 to 53.7% in 2017 (Fig. 1). The adjusted regression analysis indicated that each year during the study period was associated with an 8% increase in the proportion of EPTB among TB patients (adjusted prevalence ratio [aPR] = 1.08, 95% CI 1.03, 1.12).

**Fig. 2:**
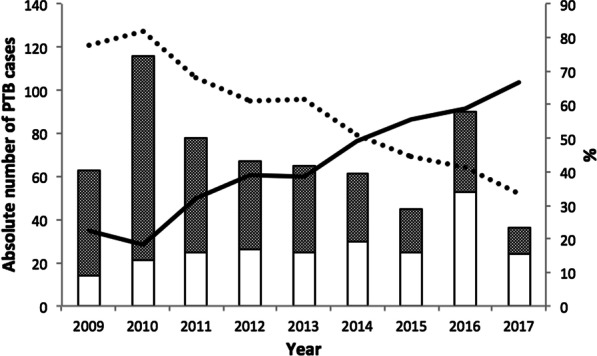
Pulmonary tuberculosis diagnosis at Kality Federal Prison, Ethiopia, 2009–2017. The annual numbers of notified pulmonary tuberculosis cases among Kality Federal prisoners (n = 621) during 2009–2017 are shown in the bar chart, stratified by pulmonary TB diagnosis (clinically diagnosed/microbiologically confirmed). The shaded bar represents clinically diagnosed cases. The line graph provides the proportion of clinically diagnosed pulmonary TB (dotted line) and microbiologically confirmed pulmonary TB (solid line) cases respectively

From 2009 to 2014, the proportion of clinically diagnosed cases of PTB remained higher than the proportion of microbiologically confirmed cases of PTB among PTB patients. During the study period, 2009–2017, the proportion of microbiologically confirmed PTB cases increased from 22.2% in 2009 to 66.7% in 2017, while the proportion of clinically diagnosed PTB cases declined from 77.8% in 2009 to 33.3% in 2017 (Fig. 2). The adjusted regression analysis indicated that each year during the study period was associated with a 15% increase in the proportion of microbiologically confirmed PTB among PTB patients (aPR = 1.15, 95% CI 1.11, 1.20).

**Fig. 3:**
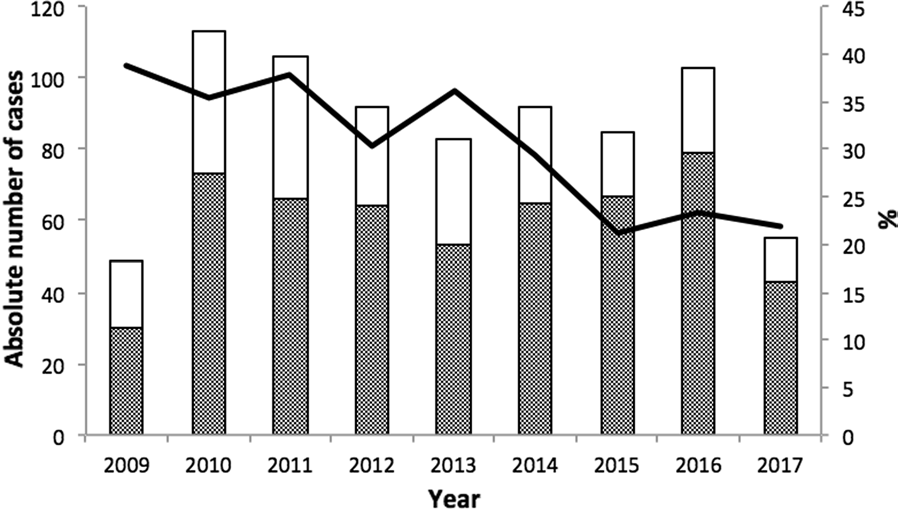
HIV sero-positivity among tuberculosis cases at Kality Federal Prison, Ethiopia, 2009–2017. The annual numbers of notified tuberculosis cases with a recorded HIV result (n = 778) at Kality Federal Prison, Ethiopia during 2009–2017 are shown in the bar chart, stratified by HIV sero-positivity (positive/negative). The positive cases are left unshaded in the bar graph. The line graph provides the proportion of HIV patients among TB cases with a recorded HIV test result

During the study period, 2009–2017, the proportion of HIV sero-positive cases among TB cases for which the HIV sero-positivity data was available declined from 38.8% in 2009 to 21.8% in 2017 (Fig. 3). The adjusted regression analysis indicated that each year during the study period was associated with a 4% decrease in the proportion of TB-HIV co-infection (aPR = 0.96, 95% CI 0.91, 1.00).

### Patient characteristics associated with EPTB

The likelihood of EPTB was 74% (aPR = 1.74; 95% CI 0.97, 3.13) and 75% (aPR = 1.75; 95% CI 0.98, 3.12) higher among 18–24 year and 25–44 year age groups, respectively, compared to 45–64 year age group (Table 2). The proportion of EPTB was lower among those with a previous TB diagnosis compared to newly diagnosed (aPR = 0.42, 95% CI 0.25, 0.70). Among TB-HIV coinfected individuals, the likelihood of EPTB was 27% lower compared to HIV sero-negative TB patients (aPR = 0.73, 95% CI 0.55, 0.97).

**Table 2:** Demographic/clinical characteristics associated with EPTB as determined by multivariable log binomial regression analysis of 736 TB cases diagnosed at Kality Federal Prison, Ethiopia, during 2009–2017

Characteristics	EPTB		
	Adj. PR	95% (CI)	*P* value^a^
Age			
18–24	1.74	(0.97, 3.13)	0.06
25–44	1.75	(0.98, 3.12)	0.06
45–64	Ref	Ref	
Previous history of TB			
Yes	0.42	(0.25, 0.70)	< 0.01
No	Ref	Ref	
HIV sero-positivity			
Positive	0.73	(0.55, 0.97)	0.03
Negative	Ref	Ref	

*Adj. PR* adjusted prevalence ratio, *CI* confidence interval

^a^*P* value from Wald Chi Square test

### Patient characteristics associated with having microbiologically confirmed PTB

The likelihood of having microbiologically confirmed PTB was similar among all age groups compared to the oldest age group (Table 3). The likelihood of having microbiologically confirmed PTB was similar among previously and newly diagnosed TB patients. The likelihood of microbiologically confirmed PTB was 22% lower among HIV sero-positive PTB patients compared to HIV-sero negative PTB patients (aPR = 0.78, 95% CI 0.61, 1.00).

**Table 3:** Demographic/clinical characteristics associated with having microbiologically confirmed PTB as determined by multivariable log binomial regression analysis of 511 TB cases diagnosed at Kality Federal Prison, Ethiopia during 2009–2017

Characteristics	Microbiologically confirmed PTB		
	Adj. PR	95% CI	*P* value^a^
Age			
18–24	1.03	(0.74, 1.43)	0.87
25–44	1.03	(0.75, 1.41)	0.87
45–64	Ref	Ref	
Previous history of TB			
Yes	1.10	(0.87, 1.38)	0.42
No	Ref	Ref	
HIV sero-positivity			
Positive	0.78	(0.61, 1.0)	0.05
Negative	Ref	Ref	

*Adj. PR* adjusted prevalence ratio, *CI* confidence interval

^a^*P* value from Wald Chi Square test

### Patient characteristics associated with TB-HIV coinfection

The likelihood of TB-HIV coinfection was 58% lower among the youngest (18–24 years) age group compared to the 45–64 year age group (Table 4, aPR = 0.42; 95% CI 0.28, 0.62). The 25–44 years age group had a similar likelihood of coinfection compared to the oldest age group (aPR = 1.08; 95% CI 0.81, 1.44). The likelihood of TB-HIV coinfection among those with a previous history of TB was 37% higher than newly diagnosed TB patients (aPR = 1.37; 95% CI 1.10, 1.71). The likelihood of coinfection among clinically diagnosed pulmonary TB cases was 1.32 times the likelihood of coinfection among microbiologically confirmed pulmonary TB cases (aPR = 1.32; 95% CI 1.02, 1.72).

**Table 4:** Demographic/clinical characteristics associated with being HIV positive as determined by multivariable log binomial regression analysis of 736 TB cases diagnosed at Kality Federal Prison, Ethiopia during 2009–2017

Characteristics	Being HIV positive		*P* value^a^
	Adj. PR	(95% CI)	
Age			
18–24	0.42	(0.28, 0.62)	< 0.01
25–44	1.08	(0.81, 1.44)	0.60
45–64	Ref	Ref	
Previous history of TB			
Yes	1.37	(1.10, 1.71)	0.01
No	Ref	Ref	
TB diagnosis			
CD-PTB	1.32	(1.02, 1.72)	0.04
EPTB	0.91	(0.65, 1.25)	0.55
MC-PTB	Ref	Ref	

*Adj. PR* adjusted prevalence ratio, *CI* confidence interval, *CD-PTB* clinically diagnosed pulmonary tuberculosis, *MC-PTB* microbiologically confirmed PTB

^a^*P* value from Wald Chi Square test

## Discussion

Population and region-specific interventions are required to achieve global elimination as contextual factors vary across place and sub-populations. Our study provides an in-depth understanding of the prison TB epidemiology, together with its interaction with the HIV epidemic, at the Kality Federal Prison, Ethiopia during 2009–2017. As the Kality Prison is not only a long term detention center but also a short term detention center, it can transmit the TB infection to the general population through short term prisoners upon release and to other federal prisons when inmates are transferred from Kality Prison [13].

The trends analyses indicated that over time the TB epidemic has evolved within the prison setting with implications for TB control and prevention management. The proportion of EPTB and microbiologically confirmed PTB increased over time while TB-HIV coinfection declined over the course of the study. These data suggest that the TB control strategy for this prison population should be refocused to include traditional, as well as non-traditional risk groups, especially based on age, previous TB diagnosis and finer TB categorizations as discussed in detail below.

The observed increase in microbiologically confirmed cases of PTB, consistent with the national trend shown in Additional file 1: Figure S1, might suggest increased risk of transmission within the prison, disease severity due to delayed diagnosis/treatment or reactivation due to prison conditions, raising concern over existing TB prevention/control strategies. However, it is also possible that these trends are reflective of changes in diagnostic practices or roll out of interventions such as Heal TB aimed at improving case detection among inmates that occurred between 2011 and 2016 [14]. Nevertheless, a recent study of the Kality Prison in Ethiopia documented that the prevalence of bacteriologically confirmed PTB (BC-PTB) was higher among jail residents compared to jail entrants, suggesting increased risk of intramural transmission or reactivation of pre-incarceration acquired PTB due to stressful prison environment [13].

Our results, although not statistically significant, suggest a 3% increase in the proportion of microbiologically confirmed PTB among 18–24 year age group, ranging between 26% reduction and 43% increase in the likelihood of microbiologically confirmed PTB (aPR = 1.03; 95% CI 0.74, 1.43). These findings may suggest lack of timely diagnosis and underutilization of medical services among this group, as 80% of the microbiologically confirmed cases in our study had a positive sputum smear result. Hence, control strategies should be focused towards these younger inmates that are presumably more likely to spread the infection, as sputum positive patients tend to be more infectious compared to only culture positive patients [15, 16]. We chose to not discredit observed effect estimates in this study solely based on *P* value threshold while also acknowledging the associated uncertainty because absence of statistical significance alone does not prove the null hypothesis [17].

A resurgence of EPTB is largely attributed to the HIV epidemic in high burden countries [18]. However, in our study, the proportion of EPTB cases increased over the course of the study period, while the HIV coinfection proportion declined. The HIV seroprevalence among TB patients was many folds higher among the prisoner population compared to the national estimates as noted by other studies as well [19–21] (Additional file 1: Figure S1). A prior study conducted among newly diagnosed HIV patients in Addis Ababa found the coinfection percentage to be 7% [22]. It appears that improved access to timely anti-retroviral treatment (ART) is driving down TB-HIV trends as HIV management in Ethiopia has shifted from providing ART to only stage 3 and 4 HIV patients or those with CD4 count < 200 cells/mm^3^ to all HIV patients irrespective of stage and CD4 count. EPTB trends are likely driven by factors other than HIV as our study did not find HIV patients to be at an increased risk of EPTB (aPR = 0.73, *P* value = 0.03). However, our sensitivity analysis (Additional file 1: Table S1) revealed that EPTB patients were more likely to have a missing HIV test result in our study (aPR = 1.94, *P* value < 0.01). If HIV sero-positive individuals among EPTB patients were more likely to not have a record for a HIV test, then it may explain the lower likelihood of EPTB among HIV positive patients compared to HIV negative patients. Various sites of EPTB may be differently associated with TB-HIV coinfection, with some such as pleural TB being negatively associated with coinfection [23]. Due to lack of precise data on EPTB site in our study population, we are not able to examine the differential associations of various EPTB sites with TB-HIV coinfection.

Additionally, our study reported that young age (18–44 years) was associated with increased risk of EPTB as compared to the older age group, although only marginally significant (*P* value = 0.06). Contrary to our finding, prior studies have reported that old age is associated with EPTB [24, 25]. This unique finding again reiterated the importance of studying the local context as traditional risk factors do not seem to explain the EPTB increasing trends. Similarly, age does not seem to alter the risk of HIV co-infection in a traditionally reported manner as the youngest group between 18 and 24 years experiences lower risk of coinfection compared to those over 45 years [26].

Prisoners having a previous TB diagnosis were more likely to have HIV coinfection, similar to earlier studies [27]. In our study, those who were lost to follow up, failed treatment or relapsed after receiving initial TB treatment were encoded as previously diagnosed TB patients. TB-HIV coinfected individuals often experience pill burden and adverse drug–drug interactions that complicate TB treatment, causing low adherence among coinfected individuals [28].

Similar to a number of previously published studies, this study also highlights the importance of resolving the TB epidemic based on its various manifestations/presentations including EPTB, microbiologically confirmed PTB, clinically diagnosed PTB and TB-HIV coinfection given that each of these TB manifestations are differentially associated with various risk factors [23, 29].

Although a previous study was conducted at the Kality Federal Prison as mentioned earlier [13], our work extends the understanding of TB epidemic among prisoners in similar endemic settings and adds to the existing literature on prison TB by including additional analyses of clinically diagnosed PTB and EPTB cases and using a much larger number of BC-PTB cases (243 vs. 22). Our study additionally assessed trends of prison TB at Kality Federal Prison to track how the TB program has performed over time while also identifying risk factors associated with EPTB, microbiologically confirmed PTB and TB-HIV coinfection. We also provide adjusted prevalence ratios (aPR) using log binomial regression rather than prevalence odds ratios (POR) from a logistic regression as PR is more interpretable and a better estimate of the true effect when the outcome is not rare [30, 31].

However, our study also had several limitations. First, due to the use of cross-sectional data, we were unable to establish a cause and effect relationship between risk factors and outcomes. We were also unable to provide incidence estimates as total population estimates for the prison were not available. The trends described in this are, therefore, not only reflective of new cases but also previously treated cases. However, 94% of the cases were new/relapse while the remaining had been previously treated or had unknown TB history. The other limitation of our study was the lack of data on the occurrence of MDR-TB due to the limited resources for routine TB diagnosis and anti-TB drug testing in the study setting. The incompleteness of data for HIV test result and comorbidity may have resulted in selection bias and residual confounding, respectively.

## Conclusion

The findings of the present study suggest that associations found between patient characteristics and different types of TB in the prisoner population may not always be consistent with previously reported associations for the general population, necessitating the need to refocus TB control/prevention strategies to target high-risk groups based on demographic/clinical factors such as young age, microbiologically confirmed pulmonary TB and previous TB diagnosis in a prison setting if WHO End TB Strategy goals are to be realized to achieve global elimination.

## Supplementary Information

**Additional file 1.** **Figure S1:** National trends for Ethiopia based on WHO notification data and **Table S1:** Demographic/clinical characteristics associated with missing HIV test result as determined by multivariable log binomial regression analysis of 871 TB cases diagnosed at Kality Federal Prison, Ethiopia during 2009–2017.

## Data Availability

All the data generated from this study are included in this published article and its supplementary information files. The datasets used and analyzed during the current study are available from the corresponding author on reasonable request and with the permission of the Kality Prison Administration, Ethiopia.
