# Supplementary material for: Patient characteristics associated with different types of prison TB: an epidemiological analysis of 921 TB cases diagnosed at an Ethiopian prison
Source: BMC Pulm Med. 2021 Oct 27;21:334. doi: 10.1186/s12890-021-01699-w (PMC8555052; doi:10.1186/s12890-021-01699-w)
Supplement: Supplementary file 1 — Additional file 1. Figure S1: National trends for Ethiopia based on WHO notification data and Table S1: Demographic/clinical characteristics associated with missing HIV test result as determined by multivariable log binomial regression analysis of 871 TB cases diagnosed at Kality Federal Prison, Ethiopia during 2009–2017. [file 12890_2021_1699_MOESM1_ESM.docx]

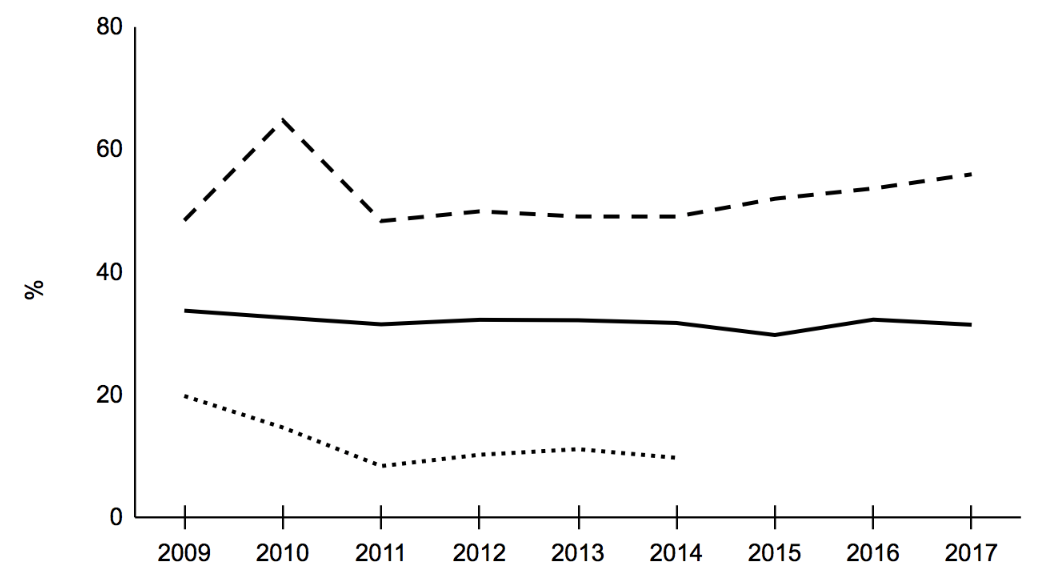


Supplementary Figure S1: National trends for Ethiopia based on WHO notification data

The extrapulmonary TB cases expressed as a percentage of total new/relapse cases is indicated by the solid line, the microbiologically confirmed pulmonary TB cases expressed as a percentage of pulmonary TB cases are indicated by the dashed line and HIV seroprevalence among those with a recorded HIV test result is indicated by the dotted line.

Supplementary Table S1: Demographic/clinical characteristics associated with missing HIV test result as determined by multivariable log binomial regression analysis of 871 TB cases diagnosed at Kality Federal Prison, Ethiopia during 2009-2017

| Characteristics |  | Missing HIV test result |  |
| --- | --- | --- | --- |
|  | Adj.PR | 95% CI | ^a^P-value |
| Age |  |  |  |
| 18-24 | 0.73 | (0.40, 1.34) | 0.32 |
| 25-44 | 0.91 | (0.51, 1.62) | 0.74 |
| 45-64 | Ref | Ref |  |
| Previous history of TB |  |  |  |
| Yes | 0.43 | (0.21, 0.86) | 0.02 |
| No | Ref | Ref |  |
| TB Diagnosis |  |  |  |
| CD-PTB | 1.22 | (0.77, 1.96) | 0.40 |
| EPTB | 1.94 | (1.26, 2.99) | <0.01 |
| MC-PTB | Ref | Ref |  |

Adj.PR= adjusted prevalence ratio; CI=confidence interval; ^a^P-value from Wald Chi Square test

CD-PTB= clinically diagnosed pulmonary tuberculosis; MC-PTB= microbiologically confirmed PTB
